# Peripheral Branch Injury Induces Oxytocin Receptor Expression at the Central Axon Terminals of Primary Sensory Neurons

**DOI:** 10.3390/ijms25010007

**Published:** 2023-12-19

**Authors:** Heni El Heni, Péter Bátor Kemenesi-Gedei, Laura Pálvölgyi, Ivett Dorina Kozma-Szeredi, Gyöngyi Kis

**Affiliations:** 1 Department of Physiology, Albert Szent-Györgyi Medical School, University of Szeged, 6720 Szeged, Hungary; 2 Department of Physiology, Anatomy and Neuroscience, Faculty of Science and Informatics, University of Szeged, 6720 Szeged, Hungary

**Keywords:** oxytocin receptor, nerve injury, dorsal root ganglia, spinal cord, gene expression

## Abstract

Considerable evidence suggests that oxytocin, as a regulatory nonapeptide, participates in modulatory mechanisms of nociception. Nonetheless, the role of this hypothalamic hormone and its receptor in the sensory pathway has yet to be fully explored. The present study performed immunohistochemistry, enzyme-linked immunosorbent assay, and RT-qPCR analysis to assess changes in the expression of the neuronal oxytocin receptor in female rats following tight ligation of the sciatic nerve after 1, 3, and 7 days of survival. Oxytocin receptor immunoreactivity was present in both dorsal root ganglia and lumbar spinal cord segments, but not accumulated at the site of the ligation of the peripheral nerve branch. We found a time-dependent change in the expression of oxytocin receptor mRNA in L5 dorsal root ganglion neurons, as well as an increase in the level of the receptor protein in the lumbar segment of the spinal cord. A peak in the expression was observed on day 3, which downturned slightly by day 7 after the nerve ligation. These results show that OTR expression is up-regulated in response to peripheral nerve lesions. We assume that the importance of OTR is to modify spinal presynaptic inputs of the sensory neurons upon injury-induced activation, thus to be targets of the descending oxytocinergic neurons from supraspinal levels. The findings of this study support the concept that oxytocin plays a role in somatosensory transmission.

## 1. Introduction

The International Association for the Study of Pain (IASP) defines neuropathic pain as an unpleasant sensory and emotional experience associated with actual or potential tissue damage, or described in terms of such damage and as pain caused by a lesion or a disease of the somatosensory nervous system. To explore the neural mechanisms of chronic pain, multiple animal models with experimental neuropathic pain have been generated. Most neuropathic pain models are produced by causing diseases or injuries to the spinal cord or peripheral nerves [1]. Pain in animals can be assessed by examining their behavioural responses to various thermal, chemical, and mechanical stimuli. While these reactions are well described in animal models, less is known about degenerative molecular changes in the spinal cord or spinal ganglion cells.

The endocrine and central neural effects of the hypothalamic/neurohypophyseal hormone oxytocin (OT) are well defined. Over the past decade, research findings have suggested that there is a novel target of OT action, namely the pain system. Systemic administration of OT or activation of the descending oxytocinergic pathway has been shown to inhibit the transmission of nociceptive information in the spinal dorsal horn [2,3,4]. Furthermore, electrophysiological studies have indicated that OT has a considerable inhibitory effect on hyperalgesia in neuropathic rats with spinal nerve ligation. This effect has been suggested to be due to the presence of OTR in the spinal dorsal horns [5]. In addition, investigations of the analgesic mechanisms have revealed that OT may exert its antinociceptive effect via the direct or indirect (via glutamatergic interneurons) activation of inhibitory GABA-ergic neurons; thus OT restrains the transmission of nociceptive impulses from the nociceptive primary afferent fibres to the second-order neurons of the spinothalamic pathway [6,7]. The analgesic effects of OT have been demonstrated in human and animal species (mainly in rats and mice as subjects), including the inhibition of somatic and visceral nociception, which, in most cases, were reversed by oxytocin receptor (OTR) antagonists [8]. Moreover, OT has been shown to affect spinal nociceptive processing via the descending oxytocinergic hypothalamo–spinal pathway, which terminates in the upper dorsal horn of the spinal cord. Eliava et al. have found a few hypothalamic parvocellular neurons (PVNs) that release OT and project to wide-dynamic range sensory neurons in the spinal cord and alleviate acute pain. Furthermore, Eliava et al. have hypothesized the existence of an additional pain suppression pathway via the activation of magnocellular oxytocinergic neurons that exert a peripheral analgesic effect on sensory neurons of the dorsal root ganglia (DRG) [9]. In addition, recent electrophysiological studies on nociceptors arising from craniofacial structures have revealed that OT, via OTRs, inhibits peripheral-evoked neuronal activity at the level of the medullary dorsal horn [10]. 

Consequently, these studies indicate that the PVN–spinal cord–OT neural pathway might regulate the development of inflammatory hyperalgesia and neuropathic allodynia carried out in the presence of OTRs. Moreover, evidence suggests that OTRs may play a role in nociception, i.e., the mechanism of pain neurotransmission. However, there are only limited data regarding OTRs’ presence in DRG; their role and the adjustment of their expression in neuropathic conditions has not been determined. The main goal of this study was to reveal the impact of peripheral nerve injury on the expression of OTR mRNA and protein in DRG and central (spinal cord–dorsal horn) afferents.

## 2. Results

### 2.1. Immunhistochemical Detection of OTR

#### 2.1.1. Analysis of the Axonal Transport of OTR

**Figure 1:**
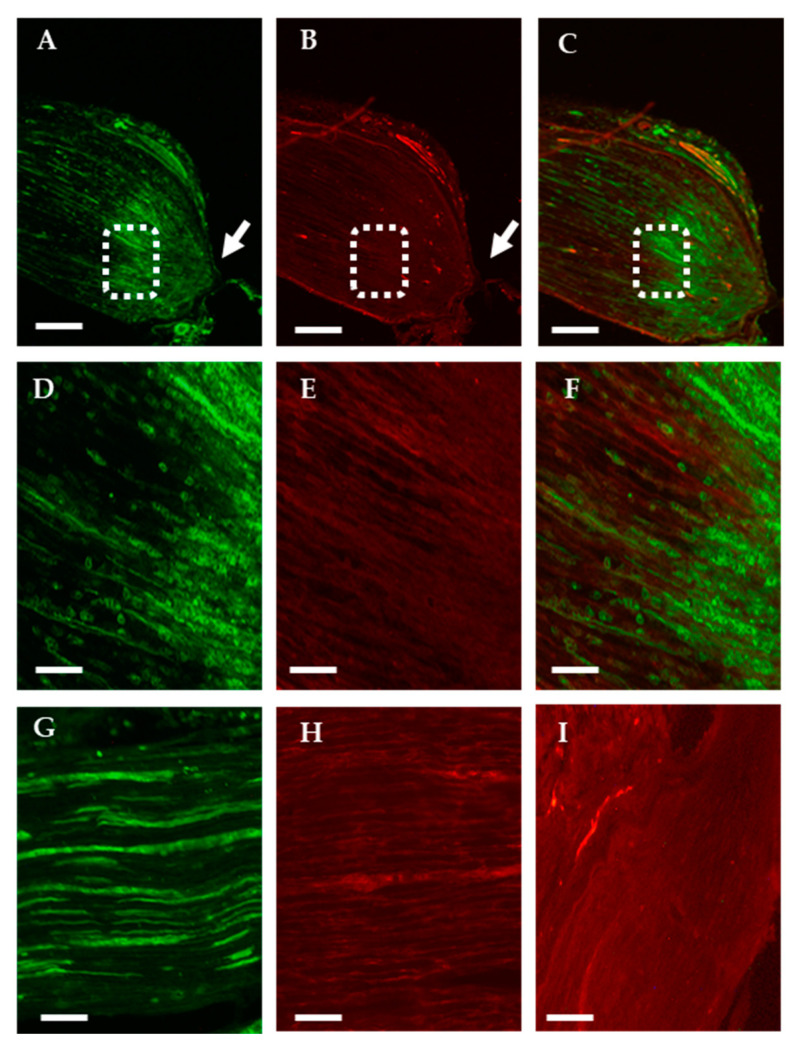
Photomicrographs represent the IB4 (green) and OTR (red) immunoreactivity in sciatic nerve sections. (**A**,**B**) show anterograde accumulation of IB4-binding glycoproteins and glycolipids (green) and that there is no accumulation of OTRs (red) proximal to the sciatic nerve ligature three days after the PNI intervention. (**D**,**E**) show the area marked by the dotted line at higher magnification. Arrows indicate the site of the tight ligature. (**G**,**H**) were obtained from contralateral nerve sections. (**C**,**F**) show the composites. In image (**I**), the negative control, only secondary antibody staining is present. Scale bars indicate 100 µm (**A**–**C**,**I**), 50 µm (**D**–**F**), and 25 µm (**G**,**H**).

We performed immunohistochemical staining on ligated nerves three days after peripheral nerve injury (PNI) intervention to detect the suggested axonal transport and accumulation of OTRs. As reference data, we observed isolectin B4 (IB4) positivity, because non-peptidergic small-diameter primary sensory neurons bind lectin B4 from *Griffonia simplicifolia*. As expected, proximity to the nerve ligation IB4 showed prominent staining in sciatic nerve tissue (Figure 1A,D), but the OTR reactivity was not greater than that of background staining (Figure 1B,E, negative control staining Figure 1I).

#### 2.1.2. Immunohistochemical Detection of OTRs in the Spinal Cord and Dorsal Root Ganglia

**Figure 2:**
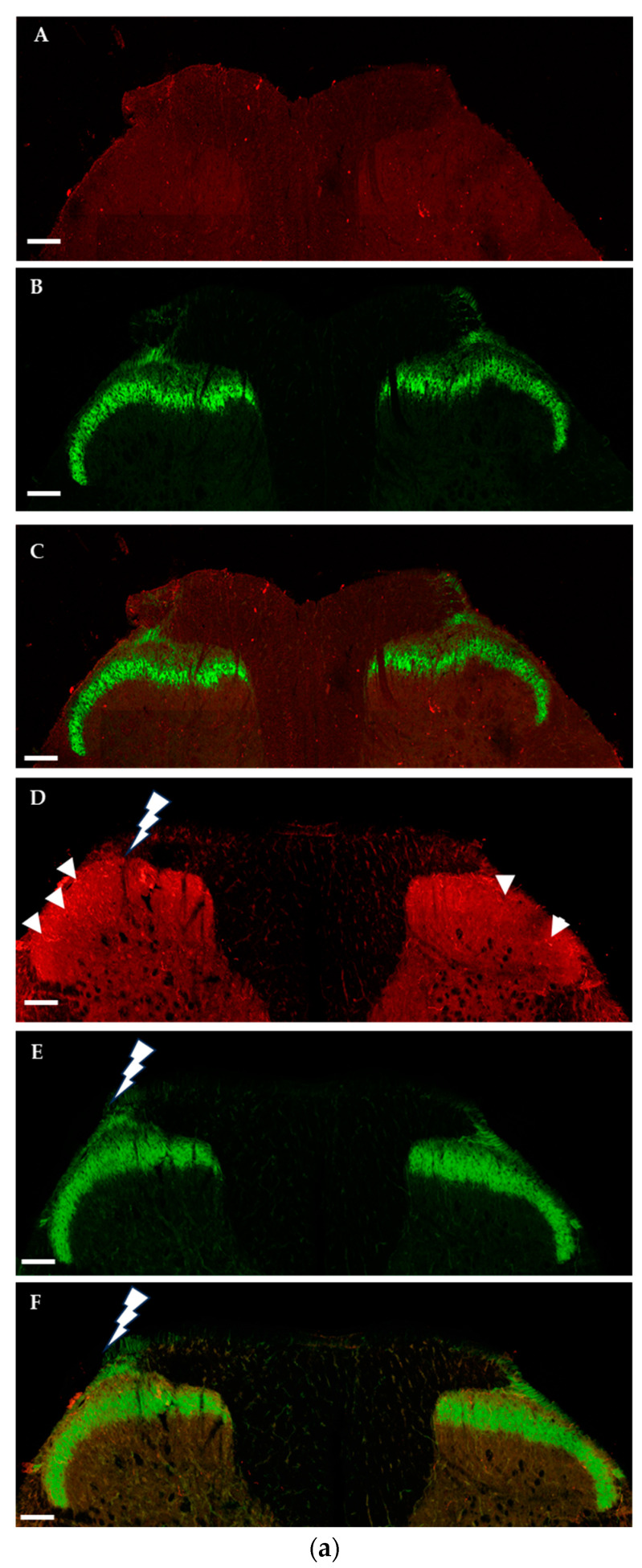

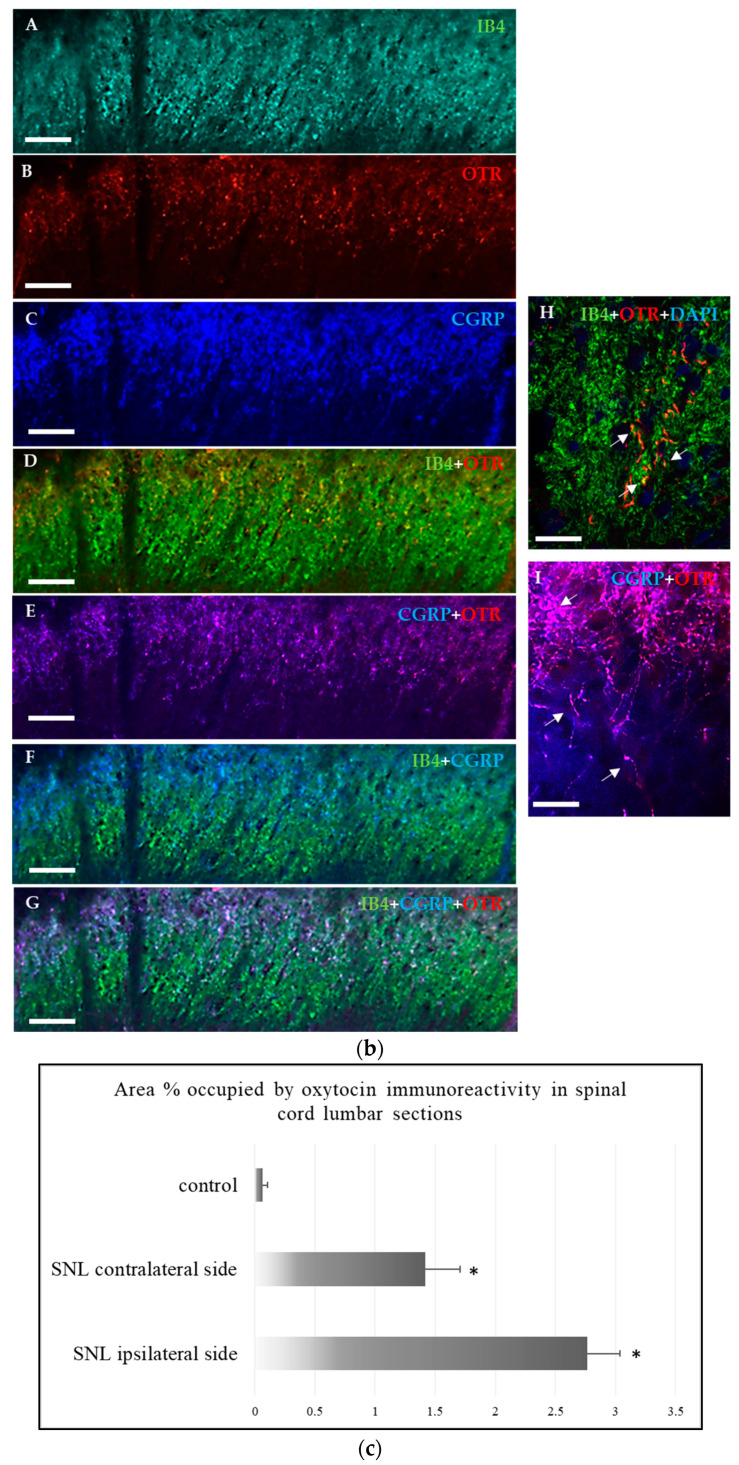
Fluorescence micrographs illustrate OTR (red) and IB4 (green) staining and composites in lumbar segment of the spinal cord dorsal horn. (**a**) (**A**–**C**) represent spinal cord from no-surgery animals and (**D**–**F**) from animals 3 days after sciatic nerve ligation, **⚡** symbols indicate the PNI sides. In figure (**D**), arrowheads point to OTR fibres in the dorsal horn superficial laminae. Scale bars indicate 200 µm. (**b**) Magnified view from PNI sides (**A**–**G**). OTR-positive fibres (red) are shown within the spinal cord dorsal horn IB4-positive layer (green) and in CGRP-positive (blue) outer laminae. Scale bars indicate 100 µm. (**H**,**I**) show colocalizations (arrows), scale bars indicate 20 µm. (**H**) contains OTR (red), IB4 (green) and DAPI nuclear staining (blue), (**I**) contains CGRP (blue) and OTR (red). (**c**) Quantitative results of proportion of area occupied by OTR immunoreactivity in the spinal cord dorsal horn grey matter, in sciatic nerve ligated animals (n = 4) compared to naïve animals (n = 4). Data are presented as mean ± SEM, * significant difference *p* < 0.001.

Immunofluorescence analysis of the OTRs in the spinal cord was performed in naïve and traumatized animals after 1, 3, and 7 days of survival periods. OTR immunoreactivity was present in the lumbar segments only after 3 days of sciatic nerve ligation and present in nerve fibres located in the superficial laminae and also in deeper laminae (Figure 2a,b). IB4 positivity indicates Rexed lamina IIi, which is substantia gelatinosa of Rolando; calcitonin gene-related peptide (CGRP) positivity is localized to the outermost layers (Figure 2b(F)), where both pain-conveying C and some A delta fibres terminate. We found OTR-positive fibre boutons colocalized with both IB4 (Figure 2b(D,H)) and CGRP (Figure 2b(E,I)). Further segments of the spinal cord were analysed, although OTR fibre immunoreactivity was observed exclusively in the lumbar level of the spinal cord, both ipsi- and contralateral to the injury (Figure 2a(D)). Quantitative analysis confirmed an increase in OTR immunoreactive area in the lumbar spinal cord 3 days after the PNI, both ipsilateral and contralateral to injury, with a larger portion occupying the ipsilateral side (Figure 2c). 

**Figure 3:**
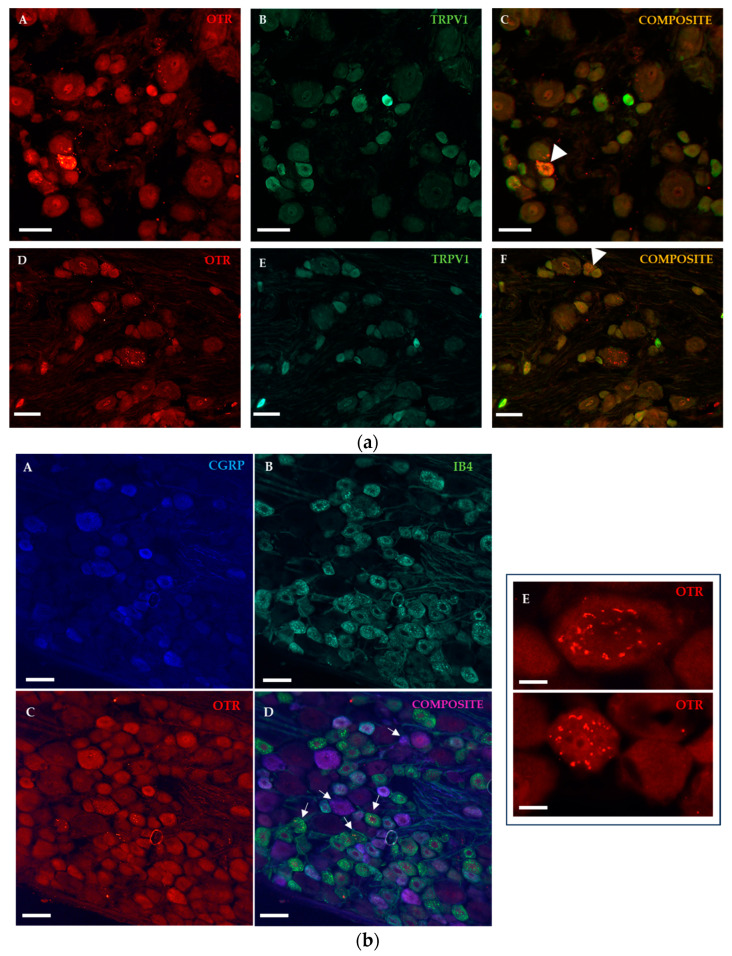
Representative fluorescence micrographs illustrate OTR-positive (red) neurons in ipsilateral L5 DRG from PNI animals with 3 days of survival. (**a**) Photomicrographs demonstrate OTR (**A**,**D**, red) and TRPV1 (**B**,**F**, green) staining. Arrowheads point to colocalization in small cells in (**C**,**F**) composites. Scale bars indicate 50 µm. (**b**) Photomicrographs show immunostainings for peptidergic CGRP-positive ((**A**), blue), non-peptidergic IB4-positive ((**B**), green), and OTR-positive ((**C**), red) neurons with composite image (**D**). Arrows point to colocalization with OTR positivity. Scale bars indicate 50 µm. In image (**E**), the OTR staining pattern is shown in larger and smaller cells. Scale bars indicate 20 µm.

In the DRG, OTR positivity was observed in cell bodies, with a characteristic cytoplasmic staining pattern (Figure 3b(E)) and occasionally colocalized with the transient receptor vanilloid 1 (TRPV1) channel, which is highly expressed in sensory neurons in PNI animals with 3 days of survival (Figure 3a). Immunostaining for IB4 binding and CGRP-positivity resulted in co-expression of OTR with both non-peptidergic and peptidergic neurons (Figure 3b(A–D)). 

### 2.2. Measurement of OTR Protein Expression with ELISA

**Figure 4:**
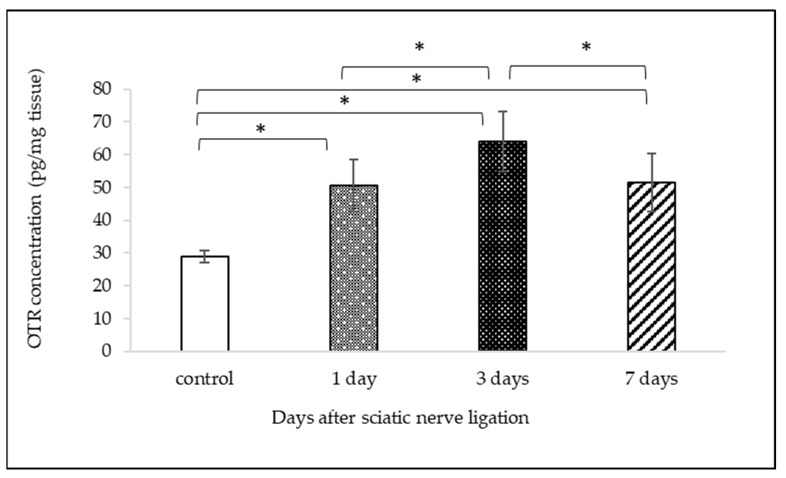
Graph shows OTR protein expression in the lumbar segments of the spinal cord after 1, 3, and 7 days after the ligation of the sciatic nerve. OTR values are expressed as pg/mg tissue. Data are presented as mean ± SD (n = 6/groups of naïve controls and survival time variants). * Significant difference (*p* < 0.001).

OTR concentrations were measured in the lumbar spinal cord homogenates. Baseline concentration of untreated control was 28.935 ± 1.77 pg/mg (mean ± SD) spinal cord tissue. Peripheral nerve ligation induced the increase of OTR levels after 1, 3, or 7 days to 50.53 ± 7.89, 64.07 ± 9.1, and 51.43 ± 8.81 pg/mg (mean ± SD), respectively (Figure 4). OTR concentrations compared to controls showed the highest elevation 3 days after the PNI, and they declined on the 7th day. The statistical analysis with one-way ANOVA indicated remarkable differences between groups (DF = 3, F = 62.814, *p* < 0.001); indeed, the Tukey post hoc test showed that the differences between all the groups were significant (*p* < 0.001), except between those with 1 and 7 days of survival (*p* = 0.937).

### 2.3. OTR mRNA Analysis with qPCR 

**Figure 5:**
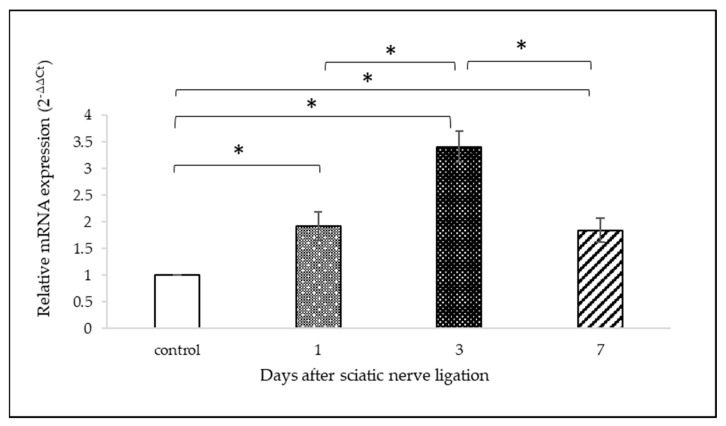
OTR mRNA level in rat L5 DRG with 1, 3 and 7 days of survival after tight ligation of the sciatic nerve. The amount of transcript level of gene in each sample is calculated using the comparative Ct method. Graph shows relative gene expressions performed on dCt values. Relative gene expression is calculated in comparison with DRG of naïve control animals, (n = 6/groups of naïve controls and survival time variants). Data are expressed as means ± SD * *p* < 0.001.

OTR gene expression in L5 DRG is presented in Figure 5. The relative changes in gene expression in animals with experimental procedure are shown over controls for each survival time. The increase in DRG OTR mRNA levels after tight ligation of the sciatic nerve was significantly elevated in all treated groups compared to DRG from intact animals. By day 3, the difference in mRNA level had reached a peak: relative gene expression was 3.41 ± 0.26-fold greater; however, only a slight decrease was observed by day 7 (1.84 ± 0.29-fold change, mean ± SD). There was a statistically remarkable difference between groups, as demonstrated via one-way ANOVA (DF = 3, F = 88.014, *p* < 0.001). A Tukey post hoc test showed that the differences between all the groups were significant (*p* < 0.001), except between those with 1 and 7 days of survivals (*p* = 0.972).

## 3. Discussion

Evidence suggests that OT plays a considerable role in pain transmission [11]. A strong analgesic effect has been shown in animal models of pain when intrathecal delivery of OT was used [12,13]. Nonetheless, prior injury to the tested tissue that causes inflammation, such as nerve ligation, is necessary for this analgesic effect [14,15]. For the trigeminal system, a similar preinjury is necessary, (i.e., ligation of the infraorbital nerve) in order for OT to inhibit the electric activity of trigeminal neurons in vitro and promote antinociception in vivo [14]. 

Neuropathic pain is an example of chronic pain with peripheral nerve injury. Pain results from the abnormal processing of a sensory input. Mechanisms that affect DRG neurons can possibly lead to a shift in the molecular supply with either disruption in the specificity of the neurochemical markers or production of new intracellular and membrane proteins of the A- and C-fibres [16,17,18,19]. The tight ligation of the sciatic nerve stops the transmission of signals (bidirectionally) along the axons, preventing muscles from working and causing loss of feeling of the area supplied by that nerve. The gathering of axonally transported cargoes, neurotransmitter vesicles, and even membrane organelles can be observed [20]. The nerve lesion we performed is a modified version of the chronic constriction injury (CCI) model developed by Bennett and Xie, as well as Medeiros et al. [21,22]. Although the CCI model causes unilateral peripheral mononeuropathy, there was considerable variance in the animals that have undergone CCI, which may have made quantitative analysis more difficult. These changes might originate from variations in the degree of the constrictions made by tying knots with sutures and from the type of suture materials as well. In our experiment, the nerve is completely transected via the ligature; thus, it is similar to the axotomy model. Peripheral ligation via interruption of axonal continuity causes substantial loss of motor, sensory, and autonomic functions that are conveyed by the traumatized nerves to the innervated body parts. Nerve fibres distal to the ligation degenerate. Compensatory mechanisms include reinnervation of the denervated targets via either regeneration of the injured axons or collateral branching of neighbouring undamaged axons and the remodelling of the nervous system circuity related to the lost functions. In addition, alterations to the activity of transcription factors and gene expression occur within a few hours after nerve trauma. Studies on neuronal injuries have revealed that there are marked changes in the expression of specific neurotransmitters, neuropeptides, ion channels, and receptors in injured neurons, as well as in the spinal cord (see reviews [17,18,19,23,24]). 

Our study focused on the involvement of OTRs in this mechanism. The expression of OTRs at the spinal level has already been demonstrated [7,9,25,26,27,28]. Accordingly, as a first step, we investigated the morphological changes that occur in the spinal cord in animals with nerve injuries. We detected OTR immunoreactive fibres in the superficial laminae of the dorsal horn and a few of the deeper laminae in the spinal cord grey matter 3 days after nerve ligation. These OTR-expressing fibres may be from targets of the descending oxytocinergic pathway from the hypothalamic level [29]; thus, the neurohormonal effect of OT on pain processing can be exerted via the reduction of the activity of nociceptive C fibres [30,31,32]. Accordingly, behavioural and electrophysiological tests performed by Godínez-Chaparro et al. have indicated that PVN stimulation selectively reduces the activity of A delta and C fibres [33]. In addition, Eliava et al. found that the parvocellular oxytocinergic neurons project to the deep layers of spinal cord, suggesting a central control by OT on a wide dynamic range of neurons in animals with inflammation-induced pain [9,27]. These reports and our findings strongly suggest that OTRs are relevant in inhibiting pain transmission via modulation of the sensory input by means of the descending hypothalamic pathways at the spinal level. Nonetheless, it was not obvious to us whether the detected OTR-positive fibres belong to interneurons or neurons raised from the spinal ganglia. In this study we demonstrated the time-dependent expression of OTR mRNA in spinal ganglion neurons and the OTR proteins in the lumbar segments of the spinal cord in animals with PNI. A remarkable increase was observed 3 days after ligation of the sciatic nerve. On day 7, a slight decrease could be seen in OTR mRNA expression as well as in protein level of the spinal cord. Similarly, Tzabazis et al. have shown that OTR expression increases when induced by peripheral inflammation in the trigeminal ganglia [34], and further studies have reported that peripheral nerve injury promotes the expression of several genes, and that the enhancement of the expression is stimulus dependent [35,36,37]. Furthermore, we demonstrated, in agreement with previous studies, that OTRs were detected both in IB4-binding non-peptidergic and CGRP-expressing peptidergic cells [7,38]. Moreover, our immunohistochemical experiments showed that the majority of the OTR-positive neurons are medium-sized or larger cells, even those with TRPV1 immunoreactivity; therefore, they presumably belong to the A delta subpopulation, in which fibres project into both the superficial and deeper layers, where they synapse with the interneurons and therefore transmit warm, cold, itch, and pain stimuli [39]. Accordingly, we assume that the OTRs present in fibres in superficial laminae originates from DRG neurons. Based on this suggestion, after tight ligation of the peripheral branch, we expected OTR accumulation proximal to the ligature, similar to how protein amassing was observed by Averill et al. [16]. However, our results failed to demonstrate unequivocal axonal accumulation of the OTR protein, in either the intact or the ligated sciatic nerve; therefore, we hypothesize axonal mRNA transport and local axonal mRNA translation and protein synthesis occurred in the central terminals of DRG neurons [40,41] in response to harmful stimulus, as has been evidenced by Bi et al. stating that the G-protein coupled opioid receptor mRNA is translocated to the axon terminals, where presynaptic translation can occur [42]. The importance of the presynaptic presence of OTRs may be explained by electrophysiological studies showing that the antinociceptive effects of OT are mediated by increased inhibitory synaptic tone and reduced neuronal firing in lamina II, which is associated with important OTR-dependent inhibition of potassium ion currents [34,35]. Considering the molecular mechanism of OT action, it has been reported that OT via OTRs can produce a membrane depolarization via altered potassium and sodium ion permeability, which increases neuronal activity in the substantia gelatinosa [36]. In addition, Gong et al. have shown that pain-sensing DRG neuron excitability is reduced by exogenous OT and causes hyperpolarization via the Ca^2+^/nNOS/NO/K^+^_ATP_ pathway [37]. These findings correlate with a study reporting that OT inhibits the firing of nociceptive neurons, stimulated electrically from the periphery, in the dorsal horn of the medulla by directly activating OTRs [10]. Accordingly, in agreement with the above-mentioned studies, we suppose that due to the presence and the injury-induced elevation in expression of OTRs, subsequently the OTR-expressing fibres take part in the coordination of antinociceptive effect of OT via presynaptic modulation induced via inputs from descending oxytocinergic axonal tracts. 

## 4. Materials and Methods

### 4.1. Animals 

Adult Wistar female rats (nulliparous, nonpregnant, nonlactating) weighing 200–250 g were maintained on a 12:12 h light/dark cycle (lights at 6:00 am) and constant temperature (21 ± 1 °C). Food and drink were available ad libitum throughout the experiment. All animals have a minimum acclimation period of 2 weeks prior to the experiments. All experiment procedures were approved by the Ethics Committee for Animal Care of the University of Szeged in accordance with the European Communities Council Directive of 24 November 1986 (86/609/EEC) under the identifier XIV/2970/2016. All efforts were made to minimize the number of animals used and their suffering. A total number of 56 animals were used as follows: n = 6/groups of naïve controls and survivals (1, 3 and 7 days) for mRNA measurement and n = 6/groups of naïve controls and survivals (1, 3 and 7 days) for protein measurement with ELISA and n = 4/groups of naïve controls and n = 4/groups of sciatic nerve ligated animals with 3 days of survival for immunohistochemistry.

### 4.2. Peripheral Nerve Injury (PNI) 

The peripheral injury model was established via constriction of the sciatic nerve based on Bennett and Xie’s model (four loose ligatures) and Medeiros et al. (one loose ligature) [21,22,43] with one modification. The sciatic nerve on the right side was tightly ligated with a cotton thread at approximately 1 cm proximal to its trifurcation, where the sural, peroneal, and tibial nerves branch off. Rats (n = 6/groups of naïve controls and survivals: 1, 3, and 7 days) were subjected to nerve ligation under anaesthesia with intraperitoneal (ip) administration of ketamine/xylazine mixture (72/8 mg/bodyweight kg) [44].

### 4.3. Immunohistochemistry (IHC)

Rats (n = 4) were deeply anaesthetized with pentobarbital (45 mg/bodyweight kg, ip) [44] and perfused through the heart with 0.9% NaCl, followed by 4% paraformaldehyde (PFA) in 0.1 M phosphate buffer (PB), pH 7.4. The spinal cord lumbar sections, the L5 dorsal root ganglia, and the sciatic nerve sections were then removed and stored in vials containing the same fixative for an additional 3 h and cryoprotected for 2 days in 0.1 M PB containing 30% glucose. The fixed tissue samples were cut in a freezing microtome at 16 µm (ganglia and nerve sections), 25 µm (spinal cord cross section), and 10 µm (spinal cord horizontal section) intervals and stored in 0.1 M PB, pH 7.4 until experimental use, which occurred 2 days later. Phosphate buffer saline (PBS) solution containing 0.3% Triton X-100 was used as solvent for all the immunohistochemical processes. After incubation with 1% normal donkey serum (NDS) for 20 min, the spinal cord sections were incubated with anti-OTR antibody (1:400, Santa Cruz Biotechnology (Dallas, TX, USA), cat.no. SC-8103) for 24 h at R.T., and then with Cy3-conjugated donkey anti-goat secondary antibody (1:500, Jacksons ImmunoResearch (West Grove, PA, USA), code no. 705-165-147) for 2 h at R.T. to detect OTRs; FITC-conjugated isolectin *G. simplicifolia* IB4 (1:500, Sigma (St. Louis, MO, USA), product no. L2895) was included with the secondary antiserum; staining with only secondary antibody was performed as a negative control. The 16 µm-thick DRG sections were prepared via cryostat microtome and incubated under the same conditions as mentioned above for NDS and antibodies to react with OTR, supplemented by anti-TRPV1 polyclonal antibody (1:500, Alomone (Jerusalem, Israel), product no. ACC-030) or anti-CGRP monoclonal antibody (1:500, Sigma, product no. C7113) and as primary and anti-rabbit Alexa Fluor^®^488-conjugated antibody (1:500, Jacksons Immunoresearch, code no. 711-545-152) or anti-rabbit Alexa Fluor^®^488-conjugated antibody (1:500, Jacksons Immunoresearch, code no. 711-545-152) or anti-mouse DyLight™ 405-conjugated antibody (1:500, Jacksons Immunoresearch, code no. 715-475-150) as a secondary antibody supplemented by FITC-conjugated isolectin *G. simplicifolia* IB4 (1:500, Sigma, product no. L2895). All sections were washed in 0.1 M PBS twice for 5 min between each incubation period and at the end mounted on glass slides and covered with ProLong™ Gold Antifade Mountant with DNA Stain DAPI (Invitrogen™ (Waltham, MA, USA), Thermofisher Scientific (Waltham, MA, USA), cat. no. P36935). The corresponding negative control experiments were carried out via incubation with secondary antibodies. 

Images were captured with a Leica DMLB fluorescence microscope (Wetzlar, Germany) equipped with a Retiga 2000R digital camera (QImaging, Surrx, BC, Canada) and Zeiss LSM 700 confocal laser scanning microscope (Carl Zeiss Microscopy GmbH, Göttingen, Germany) using ZENversion 2.1 software (Carl Zeiss Microscopy GmbH, Göttingen, Germany). A random selection of spinal cord sections (2 from each animal) of the L5 spinal cord segment was made, and the OTR immunoreactivity was analysed. The regions of interest were determined using IB4 positivity as an orientation aid that demarcates Rexed lamina IIi. The proportion of areas was calculated using ImageJ software version 1.54f (NIH, Bethesda, MD, USA) according to Hartig [45] and the values from 2 sections per animals (n = 4/group of PNI with 3 days of survival and n = 4/naïve control group) were averaged to obtain the value of the OTR immunoreactive area. 

### 4.4. Enzyme-Linked Immunosorbent Assay (ELISA)

The rats (n = 6/groups of naïve controls and survivals: 1, 3, and 7 days) were deeply anaesthetized with pentobarbital (45 mg/bodyweight kg, ip) [44] and after exsanguination 40 mg portions of the spinal cord were dissected from where dorsal roots of the L5 ganglia terminated. The samples were rinsed in ice-cold PBS (0.01 M, pH 7.4), blotted, and weighed, and then immediately placed in liquid nitrogen. On analysis, the tissue samples were homogenized in PBS (0.01 M, pH 7.4) on ice with a plastic homogenizer and sonicated with an ultrasonic cell disrupter for 5 min. Protein measurement was carried out using a commercial ELISA kit (FineTest, Wuhan, China), cat.no.ER 1619) according to the manufacturer’s protocol. Briefly, the OTR content was extrapolated from a standard curve that was produced by the diluted assay standard. Correction for sample weight occurred as follows: the OTR content in the ELISA wells (obtained in pg/mL) multiplied by the ratio of total lysate volume divided by the appropriate tissue weight (obtained in pg/mg). Data are presented in pg/mg tissue spinal cord section weight.

### 4.5. RNA Extraction and Quantitative Real-Time Polymerase Chain Reaction (qRT-PCR)

qRT-PCR was used to detect the amount of targeted mRNAs in L5 dorsal root ganglia (DRG) after 1, 3, and 7 days of tight ligation of the sciatic nerve. Before exsanguination and the removal of the L5 DRG, the rats (n = 6/groups of naïve controls and survivals: 1, 3, and 7 days) were deeply anaesthetized with pentobarbital (45 mg/bodyweight kg, ip) [44]. RNA extraction was performed according to the manufacturer’s instructions using TriXtractTM reagent (G-Biosciences (St. Louis, MO, USA), cat. no. 786-652). After homogenization of tissue samples in TriXtractTM reagent, the RNA content was separated into an aqueous phase with the addition of chloroform. The precipitation with isopropyl alcohol was followed by a washing with 70% ethanol and then the RNA pellet was dissolved in RNase-free water. The quantity and quality were verified using Genova Nano micro-volume spectrophotometer (Jenway, Chicago, IL, USA) at an optical density of 260 and 260/280 nm, respectively; all samples that were used for further analysis exhibited an absorbance ratio in the range of 1.6–2.0. Equal amounts of the RNA were used to synthetize cDNA in each experiment using iScript cDNA synthesis kit (Bio-Rad (Hercules, CA, USA) cat.no. 1708891). The control L5 DRG from the left side were processed together as a single sample, whereas the L5 DRG from the treated side were processed separately from the animal. PCR was carried out in a thermo cycler (Bio-Rad CFX96TM Optics Module) preparing triplicates of reactions of 10 µL in volumes using iQ™ SYBR^®^ Green Supermix (Bio-Rad, cat.no. 1708882). The thermal cycling condition included an initial denaturation step at 95 °C for 30 s and 40 cycles of denaturation at 95 °C for 10 s, annealing at 57 °C for 30 s, and extension at 72 °C for 20 s. Finally, the amplicons were subjected to melting curve analysis. A pair of primers previously designed Bangaru et al. was applied in-house to amplify a 129 bp fragment of the mitogen-activated protein kinase 6 (MAPK6) mRNA [46]. Expression of MAPK6 as housekeeping gene (loading control) was determined from the same set of samples to use it as internal normalizer. Another pair of primers was designed using the National Centre of Biotechnology Information (NCBI) reference sequence database (https://www.ncbi.nlm.nih.gov/Entrez, accessed on 19 May 2020) to amplify a 140-bp fragment of the OTR mRNA (Table 1). A no-template was used as negative control where RNAse-free water was added instead of cDNA. The threshold cycle values (Ct) were used as reference points for calculating relative gene expression. The comparative Ct method, also known as Δ∆Ct method [47] was implemented to achieve relative quantification. 2^−Δ∆Ct^ values were used to calculate fold changes in target gene expression using control groups as normalizers. 

### 4.6. Statistical Analysis

To analyse the results and to determine the differences between the PNI and naïve groups for the oxytocin fibre immunoreactivities in the spinal cord sections, a pairwise comparison (Student’s *t*-test) was used. Differences were considered statistically significant at *p* < 0.05. 

Statistical comparisons were conducted using one-way analysis of variance (ANOVA), followed by Tukey’s post hoc test to compare oxytocin receptor protein and mRNA expressions of naïve animals to those with PNI of different survival days. Differences were considered statistically significant at *p* < 0.05. 

## 5. Conclusions

In sum, this study contributes to the better understanding of the mechanisms of the recently described peripheral antinociceptive effects of oxytocin. Here we presented that the anatomical substrates of the antinociceptive action of oxytocin, i.e., the primary afferent neurons express OTRs, and both the mRNA and protein levels of the receptor are elevated under in vivo conditions in the neuronal bodies and the spinal cord, respectively, following peripheral nerve lesion. Beyond that, OTR protein expression is likely to occur in the central axon terminals, but more thorough examination is necessary to secure this theory. Based on previous studies and our present results, we assume that the importance of OTRs is to modify spinal presynaptic inputs upon activation and thus for them to be targets of the descending oxytocinergic neurons from the supraspinal systems. This study is consistent with the view that oxytocin may have a role in the ascending somatosensory pathway.

## 6. Limitations

### 6.1. Choice of the Antibody

The specificity of the antibody was assessed by performing immunostaining controls to avoid false positive OTR signal detections. The lack of primary antibody during the immunostaining procedure failed to show specific OTR staining. In addition, the same antibody was used by several different research groups. As first, Moreno-Lopez et al. identified the OTR-positive nociceptive neurons in DRG, later, Gonzalez-Hernandez et al. have also published an article in which they reported the presence of OTRs in cutaneous peptidergic terminals, and finally, Tzabazis et al. have used this antibody to stain trigeminal primary sensory neurons [7,27,38]. 

### 6.2. Choice of Female Rats 

Oxytocin is traditionally thought to be a female neurohypophysis hormone that has an important role in parturition and milk ejection. However, Severino et al. has found no sex difference either in the oxytocin signalling or the recovery after spinal nerve ligation [48]. We used nulliparous, nonpregnant, nonlactating female animals during the experiments. The oxytocin content of the ovaries alters with the phases, and the plasma levels of oxytocin have not shown significant differences among the various phases of the oestrous cycle [49], hence, we did not evaluate the oestrus cycles of the rats.

## Figures and Tables

**Table 1:** Primer sequences used for mRNA expression studies.

Target Gene	Primer Pairs (5′→3′)	Product Length	Reference Sequence
Mitogen-activated protein kinase 6 (MAPK6)	Fw: TAAAGCCATTGACATGTGGG ^1^ Rev: TCGTGCACAACAGGGATAGA ^1^	129	NM_031622.2
Oxytocin receptor (OTR)	Fw: TTCTTCTGCTGCTCTGCTCGT Rev: TCATGCTGAAGATGGCTGAGA	140	NM_012871.3

^1^ Obtained from Bangaru et al. [46].

## Data Availability

The data that support the findings of this study are available from the corresponding author G.K., upon reasonable request.
